# High-normal estimated glomerular filtration rate and hyperuricemia positively correlate with metabolic impairment in pediatric obese patients

**DOI:** 10.1371/journal.pone.0193755

**Published:** 2018-03-05

**Authors:** Roberta Ricotti, Giulia Genoni, Enza Giglione, Alice Monzani, Martina Nugnes, Sara Zanetta, Matteo Castagno, Agostina Marolda, Giorgio Bellomo, Gianni Bona, Simonetta Bellone, Flavia Prodam

**Affiliations:** 1 SCDU of Pediatrics, Department of Health Sciences, University of Eastern Piedmont, Novara, Italy; 2 Clinical Chemistry, Department of Health Sciences, University of Eastern Piedmont, Novara, Italy; 3 Interdisciplinary Research Center of Autoimmune Diseases (IRCAD) University of Eastern Piedmont, Novara, Italy; 4 Endocrinology, Department of Translational Medicine, University of Eastern Piedmont, Novara, Italy; University of Colorado Denver School of Medicine, UNITED STATES

## Abstract

**Background:**

Childhood obesity represents a major health concern worldwide due to its well established detrimental effect on cardiovascular and its potential negative effect on kidney functions. However, biomarkers that can help diagnose early stages of kidney damage in obese children represent an unmet clinical need.

**Objectives:**

In this study, we asked whether the prevalence of microalbuminuria, estimated glomerular filtration rate (eGFR) or hyperuricemia recorded in a wide cohort of obese children and adolescents would positively correlate with cardiometabolic dysfunction in these subjects.

**Methods:**

We carried out a cross-sectional study on 360 obese children and adolescents between the ages of 3–18 years, enrolled in a tertiary care center. Clinical and biochemical evaluations including oral glucose tolerance tests (OGTTs) were performed on all patients. Microalbuminuria was defined as urinary albumin-to-creatinine ratio (u-ACR) of 30–300 mg/g. All data are expressed as mean ± standard deviation (SD), absolute values or percentages. Sex age-specific and eGFR SDs were used for statistical analyses. Serum uric acid ≥ 5.5 mg/dL was considered abnormal.

**Results:**

The prevalence of microalbuminuria was 6.4%. Except for a lower insulinogenic-index, no correlations between microalbuminuria and cardiometabolic risk factors were detected. eGFR was < -1 SD and > 1 SD in 1.4% and 60.8% of subjects, respectively. Subjects with an eGFR > 1 SD had higher systolic blood pressure, liver enzymes, insulin resistance, glucose and insulin during OGTT, lower insulin sensitivity and a more prevalent microalbuminuria. Hyperuricemia (27.5%) increased the odds of hypertension, HDL ≤ 10^th^ percentile and glucose ≥ 155.0 mg/dL after 60 minutes of OGTT.

**Conclusions:**

A worse cardiometabolic profile was observed in subjects with an eGFR > 1 SD compared to other subgroups. Therefore, pediatric obese patients with eGFR > 1 SD or hyperuricemia should be closely monitored for microalbuminuria and post-challenge glucose and insulin secretion, all potential indicators of renal dysfunction in these young patients.

## Introduction

The rising prevalence of pediatric obesity is one of the most challenging public health issues worldwide. Most of the major concerns derive from the fact that childhood obesity, besides increasing traditional cardiometabolic risk factors, can seriously hamper kidney function [1]. In this regard, the recent global obesity epidemic has coincided with a dramatic rise in the prevalence of end-stage renal disease (ESRD) [1]. To make matters worse, emerging evidence suggests that renal dysfunction may start early during childhood, long before a diagnosis of hypertension with type 2 diabetes can be made, and it might be related to pediatric obesity [1].

Since onset of the obesity-associated renal disease is generally subtle and asymptomatic, there is clearly an urgent need of biomarkers that could allow early detection of kidney dysfunction in obese children. In this regard, mounting evidence indicates that in adults the prevalence of microalbuminuria, which is often associated with nephropathy and atherosclerosis, positively correlates with the degree of obesity [2,3]. Furthermore, a relationship between microalbuminuria and obesity has also been reported in children and adolescents [1], although long-term studies in these patients have yet to be conducted. Interestingly, the association between estimated glomerular filtration rate (eGFR) and some cardiometabolic risk factors appears non-linear as either low- or high-normal eGFR has been associated with increased risk of metabolic diseases and mortality [2]. However, whether eGFR represents a *bona fide* cardiometabolic risk indicator, especially in pediatric patients, still remains to be determined [2,4].

In addition to microalbuminuria, hyperuricemia is another well-established risk factor for chronic kidney disease (CDK) in adults [5]. This is probably due to the detrimental effects exerted by uric acid once it permeates a cell, which obviously counteracts its antioxidant activity in the extracellular environment [6]. Therefore, hyperuricemia has a negative impact on both metabolism and longevity independent of traditional cardiometabolic risk factors [5]. However, data concerning hyperuricemia in obese children and adolescents are still lacking.

Here, we have conducted a cross-sectional study on a wide cohort of obese pediatric patients to determine 1) the prevalence of microalbuminuria; 2) the distribution of age- and sex-specific eGFRs; 3) the prevalence of hyperuricemia; and 4) any correlations between microalbuminuria, eGFR and uric acid and other known cardiometabolic markers.

## Materials and methods

### Study design

This was a cross-sectional study. Study quality was assessed (S1 Table). We consecutively recruited Caucasian children and adolescents, aged 3–18 years, referred to our Pediatric Endocrine Service from January 2011 to June 2014 for simple obesity. Subjects were eligible if generally healthy, overweight or obese, according to the IOTF criteria [7], and naïve to a weight-loss diet. Among children that had been previously subject to biochemical investigations due to any medical condition, only healthy children were included in the study. Subjects who refused to perform an oral glucose tolerance test (OGTT) were included only if they underwent fasting biochemical evaluations. Exclusion criteria were diagnosed or suspected endocrine or genetic obesity, type 1 diabetes and previous kidney diseases. Subjects referred to our Service for known comorbidities of obesity (e.g. glucose alterations, arterial hypertension, dyslipidemia, liver steatosis, hyperuricemia etc.) were also excluded to avoid interferences due to previous lifestyle or pharmacological interventions.

The protocol was conducted in accordance with the declaration of Helsinki and was approved by the Local Ethic Committee of AOU Maggiore della Carità of Novara (CE 95/12). Informed consents was administered to all patients and parents of minors prior to the evaluations, and the study was carefully explained by the research team to all parents and children. Only those patients who provided a written informed consent were included in the study.

### Anthropometric and biochemical measurements

Height was measured to the nearest 0.1 cm using a Harpenden stadiometer. Body weight was measured with light clothing to the nearest 0.1 kg using a mechanical column weighing scale (Wunder, weighing capacity 200 Kg). Body mass index (BMI) was calculated as body weight divided by squared height (kg/m^2^). The BMI standard deviation score (BMI-SDS) was calculated by the least median squares (LMS) method as previously described [8]. Waist circumference (WC) was measured at the high point of the iliac crest around the abdomen and was recorded to the nearest 0.1 cm. A non-elastic flexible tape was used, with the subjects being kept in a standing position with minimal respiration and no clothing covering the waist area or compressions on the skin. The waist-to-height ratio was calculated as well. Pubertal stages were determined by physical examination, using the criteria of Marshall and Tanner. Systolic BP (SBP) and diastolic BP (DBP) were measured three times at 2-minute intervals using a mercury sphygmomanometer with an appropriate cuff size after participants had been sitting quietly for at least 15 minutes, with their right arm being supported at the level of the heart, and feet resting flat on the floor, prior to other physical evaluations and at least 30 minutes after blood sampling. Mean values were used for all these analyses. Hypertension was determined only if BP values recorded at enrollment and testing day were both found elevated. After a 12-hour overnight fast, blood samples were taken for measurement of: glucose (mg/dL), insulin (μUI/mL), total cholesterol (mg/dL), high density lipoprotein (HDL)-cholesterol (mg/dL), triglycerides (mg/dL), aspartate aminotransferase (AST, IU/L), alanine aminotransferase (ALT, IU/L), uric acid (mg/dL), creatinine (mg/dL), IGF1 (ng/mL), 25-hydroxy (OH) vitamin D (ng/mL), using standardized methods in the Hospital’s Laboratory [9]. Low-density lipoprotein (LDL)-cholesterol was calculated by the Friedwald formula. AST-to-ALT ratio was calculated. Uric acid (mg/dL) was measured by the Fossati method using uricase with a Trinder-like endpoint. Serum creatinine concentration (mg/dL) was measured by the enzymatic method. Glucose was determined by the hexokinase method (Slein Method, Advia 1200/1800/2400 Autoanalyzer; Bayer Diagnostics, Leverkusen, Germany) with an intra-assay coefficient of variation of 0.7–2.3% (range 0.0 mg/dL -700.0 mg/dL). Insulin was determined by an immunoassay method (Advia Centaur®; Bayer Diagnostics, Leverkusen, Germany) with an intra- and inter-assay coefficient of variation of 3.2–4.6% and 2.6–5.9%, respectively (range 0.5 mU/L-300.0 mU/L). Urine albumin (mg/L) was determined by an advanced immunoturbidimetric assay, and urine creatinine (mg/dL) was measured using the enzymatic method.

Subjects also underwent OGTT (1.75 g of glucose solution per kg, maximum 75 g), and samples were drawn for the determination of glucose and insulin every 30 minutes. The area under the curve (AUC) was calculated according to the trapezoidal rule. Insulin resistance was calculated using the formula for homeostasis model assessment (HOMA)-IR. Insulin sensitivity at fasting and during OGTT was calculated with the formula of the Quantitative Insulin-Sensitivity Check Index (QUICKI) and Matsuda index (ISI). Insulinogenic (InsI) and disposition (DI) indexes were also calculated as previously reported [10]. The stimulus for insulin secretion in the increment in plasma glucose as the insulinogenic index was calculated as the ratio of the changes in insulin and glucose concentration from 0 to 30 minutes (InsI). Βeta-cell compensatory capacity was evaluated by the disposition index defined as the product of the ISI and InsI (DI) [11]. Glucose was expressed in mg/dL (1 mg/dL = 0.05551 mmol/L) and insulin in μUI/mL (1 μUI/mL = 7.175 pmol/L) in each formula.

### Definitions

Subjects were classified as overweight or obese according to age- and sex-specific IOTF cut-offs [7]. WC percentiles were stratified according to sex and age, identifying abdominal obesity as the presence of WC ≥ 90^th^ percentile or a waist-to-height ratio of 0.5 [10]. SBP and DBP values were evaluated according to percentiles for age, sex and height, and arterial hypertension was defined as SBP or DBP > 95^th^ percentile. Triglycerides, LDL- and HDL-cholesterol percentiles for age and sex were classified according to the Lipid Research Clinic Pediatric Prevalence Study. Dyslipidemia was defined as the presence of triglycerides ≥ 90^th^ percentile, HDL-cholesterol ≤ 10^th^ percentile or LDL ≥ 90^th^ percentile. Impaired fasting glucose and impaired glucose tolerance were defined as fasting plasma glucose ≥ 100–125 mg/dL (5.6 to 6.9 mmol/L) and 2-hour post-OGTT, glucose ≥ 140–199 mg/dL (7.8 to 11.0 mmol/L), respectively. Uric acid ≥ 5.5 mg/dL was considered abnormal [12]. According to the NKF-K/DOQI Guidelines for chronic kidney disease (CKD) in children and adolescents [13], the eGFR was calculated using the modified Schwartz’s formula [14]: eGFR (mL/min/1.73 m^2^) = [0.413 x patient’s height (cm)] / serum creatinine (mg/dL). The normal renal function of patients [mean eGFR ± standard deviation (SD) in mL/min/1.73 m^2^] was calculated based on age and gender according to NKF-K/DOQI Guidelines [13]. 2–12 year-old males and females: 133±27 mL/min/1.73 m^2^; 13–21 year-old males: 140±30 mL/min/1.73m^2^; and 13–21 year-old females: 126±22 mL/min/1.73 m^2^. Because only 6 subjects had eGFR lower or higher than ±2SD, the population was divided into four categories according to age and gender ±1SD (range: < -1 SD; -1-0 SD; 0–1 SD; > 1 SD). All subjects collected first-morning urine samples at rest. Urine albumin-to creatinine-ratio (u-ACR; mg/g) was calculated as follows: [urine albumin (mg/dL)/urine creatinine (g/dL)]. Microalbuminuria was defined as u-ACR ranging from 30 to 300 mg/g [15]. We collected two more samples from the subjects found positive for microalbuminuria to confirm the measurement. Microalbuminuria was diagnosed if all the three samples were found positive.

### Statistical analysis

All data are expressed as mean ± SD, absolute values or percentages. In the case of microalbuminuria, the u-ACR mean values of the three first-morning samples were used as continuous variables. With an expected prevalence of 14% of microalbuminuria [6], a confidence level of 99.0% and a margin of error of 5.0%, a population size of 320 individuals was estimated sufficient to reflect our target population. Skewed variables were logarithmically transformed. ANOVA was used to determine the differences among sex, the presence of microalbuminuria, hyperuricemia, and the eGFR subgroups with a Bonferroni post-hoc test for multiple comparisons in the latter. Analysis of covariance (ANCOVA) was also used for hyperuricemia and eGFR and covariates were age, sex, puberty and BMI (Model 1) or WC (Model 2), according to the significant relationship with dependent variables. Logistic regression was used to determine the association of microalbuminuria, eGFR and uric acid with the odds ratio (OR, 95% CI) of each cardiometabolic risk factor. Covariates of model 1 and 2 were also used in logistic regression for hyperuricemia and eGFR. Correlations as well as partial correlations were performed. Significance was assumed at *p* < 0.05. The analysis was carried out with SPSS for Windows version 17.0 (SPSS Inc., Chicago, IL, USA). The database of the study is available upon request for validation or collaboration purposes as it includes other data (e.g. family history and other biochemical variables) not yet analyzed.

## Results

### Anthropometric and metabolic characteristics of patients

Nineteen out of 379 subjects selected were excluded because they did not satisfy inclusion criteria (fifteen subjects did not have adequate blood sampling, and 4 were without at least 3 urine collections). The final dataset included 360 participants (180 males and 180 females), aged 3 to 18 years, with a mean age of 10.9±3.0 years. Of the 360 participants, 18 subjects did not undergo OGTT (fifteen refused, 3 had analyses missing for technical problems), but had a complete fasting biochemical evaluation. Among patients 88% of them were obese, and 12% overweight. Almost all subjects had a WC ≥ 90^th^ percentile (97.8%) with an overall mean of the waist-to-height ratio of 0.63±0.11, without differences between sexes. The clinical and biochemical characteristics of subjects are reported in Table 1. Hypertension was diagnosed in 216 (60.0%) subjects. Eighty-eight subjects (24.4%) had triglycerides ≥ 90^th^ percentile; 148 (41.1%) had HDL-cholesterol ≤ 10^th^ percentile; and 29 (8.0%) had LDL-cholesterol ≥ 90^th^ percentile. Twenty subjects (5.5%) had impaired fasting glucose, 19 (5.2%) impaired glucose tolerance and 4 (1.1%) both metabolic dysfunctions. One patient had type 2 diabetes.

**Table 1:** Clinical and biochemical features of the study population according to sex.

		Overall	M	F
**Subjects**		360	180	180
**Age (years)**		10.9±3.0	10.7±2.8	11.1±3.3
**Puberty**	**PP**	155 (43.1%)	98 (54.4%)	57 (31.7%) ^*†*^
	**P**	205 (56.9%)	82 (45.6%)	123 (68.3%) ^*†*^
**BMI (kg/m**^**2**^**)**		28.12±4.52	27.83±3.85	28.40±5.09
**Obesity IOTF**		317 (88.0%)	159 (88.3%)	158 (87.8%)
**BMI SDS (kg/m**^**2**^**)**		2.09±0.46	2.06±0.41	2.13±0.50
**Waist circumference (cm)**		90.8±13.4	90.6±12.0	91.1±14.6
**Waist/Height ratio**		0.63±0.11	0.62±0.10	0.64±0.11
**SBP (mmHg)**		126±16.1	126±16.8	126±15.3
**SBP percentile**		90±14.9	89±15.3	90±14.5
**DBP (mmHg)**		79±10.8	79±10.8	79±10.7
**DBP percentile**		87±15.3	87±15.1	87±15.3
**Total cholesterol (mg/dL)**		145.6±27.7	144.6±26.9	146.8±28.5
**HDL-c (mg/dL)**		42.8±8.7	43.1±8.6	42.5±8.7
**LDL-c (mg/dL)**		87.3±23.5	86.9±23.4	87.7±23.7
**Triglycerides (mg/dL)**		77.8±43.5	72.4±35.8	83.1±9.6 ^*^
**AST (IU/L)**		23.8±7.0	25.2±7.0	22.5±6.8 ^*†*^
**ALT (IU/L)**		24.3±13.0	26.4±15.8	22.2±8.9 ^*^
**AST/ALT ratio**		1.10±0.39	1.11±0.38	1.10±0.39
**Uric acid (mg/dL)**		4.87±1.20	4.97±1.30	4.76±1.07
**IGF-1 (ng/mL)**		286.0±129.7	259.2±129.7	313.1±124.2 ^*†*^
**eGFR (mL/min/1.73m**^**2**^**)**		119.78±19.70	120.6±19.4	118.91±19.95
**GlcT0' (mg/dL)**		87.9±7.3	88.3±7.1	87.5±7.5
**GlcT30' (mg/dL)**		134.8±22.4	136.9±23.0	132.7±22.5 ^*^
**GlcT60' (mg/dL)**		115.1±25.9	116.1±26.5	114.0±25.3
**GlcT90' (mg/dL)**		108.9±20.0	108.7±19.5	107.5±21.3
**GlcT120' (mg/dL)**		108.0±21.6	109.9±18.3	106.0±18.3 ^*^
AUC Glc (mg/dL*h/dL)		13816.4±2866.7	14035.7±3513.6	13587.8±1965.3
**Mean Glc (mg/dL)**		111.7±20.5	113.4±24.6	109.9±14.9
**InsT0' (mUI/L)**		16.4±11.4	14.7±10.0	18.1±12.4 ^*^
**Mean Ins (mUI/L)**		75.6±62.3	72.9±67.5	78.3±56.6
**HOMA-IR**		3.66±2.80	3.37±2.72	3.96±2.85 ^*^
**ISI**		4.71±4.70	4.77±3.78	4.66±5.50 ^*^
**QUICKI**		0.33±0.05	0.33±0.04	0.33±0.05 ^*^
**InsI**		2.14±4.04	2.05±4.88	2.22±2.96
**DI**		6.79±16.84	6.38±11.96	7.22±20.73
**u-ACR (mg/g)**		11.30±26.99	9.11±20.64	13.48±32.01 ^*^

Data are expressed as mean±SD. *p* value < 0.01*; < 0.0001^†^. OGTT data are available for 342 or 345 subjects. Abbreviations: ALT: alanine aminotransferase; AST: aspartate aminotransferase; AUC: area under the curve; BMI: body mass index; DI: disposition index; DBP: diastolic blood pressure; eGFR: estimated glomerular filtration rate; F: female; GlcT0’: fasting glucose; GlcT30',T60',T90',T120’: post-challenge glucose; HDL-c: high density lipoprotein cholesterol; HOMA-IR: homeostatic model assessment of insulin resistance; InsI: insulinogenic index; InsT0’: fasting insulin; IOTF: International Obesity Task Force; ISI: insulin sensitivity index; LDL-c: low density lipoprotein cholesterol; M: male; P: pubertal; PP: prepubertal; QUICKI: quantitative insulin-sensitivity check index; SBP: systolic blood pressure; u-ACR: urinary albumin-to-creatinine ratio.

### Microalbuminuria and associations between eGFR, uric acid, and other cardiometabolic variables

Microalbuminuria was detected in 6.4% (23/360) of subjects. In patients with or without microalbuminuria, uric acid was ≥ 5.5 mg/dL in 34.7% (8/23) and 27.0% (91/337) of them, respectively (p = 0.278). All patients with microalbuminuria had eGFR > 0 SD. In particular, 69.6% (16/23) of them had eGFR > 1 SD. In the entire cohort without microalbuminuria, 91.7% (309/337) had eGFR > 0 SD, and in 60.2% (203/337) of them the eGFR was > 1SD.

Subjects with microalbuminuria had lower insulin levels at 30-minute post-OGTT (81.8±72.8 mUI/L *vs* 122.3±163.5; p < 0.02) and lower insulinogenic index (InsI) (0.74±5.08 *vs* 2.24±4.04; p < 0.05) than those without it. No correlations between u-ACR as a continuous variable and cardiometabolic alterations were found.

### eGFR evaluation and its association with microalbuminuria, uric acid, and other cardiometabolic variables

**Fig 1:**
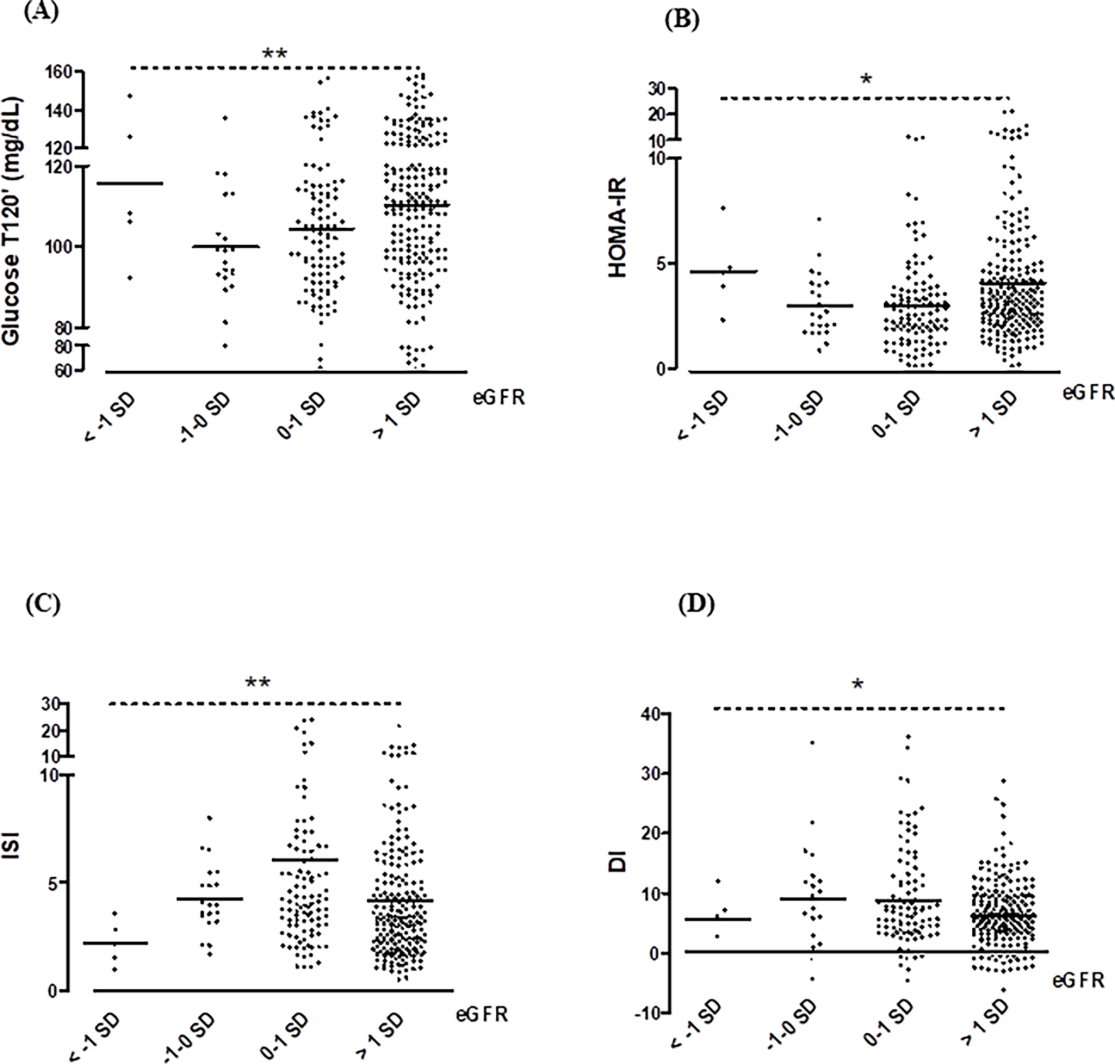
Relationship between glucometabolic parameters and stratified estimated glomerular filtration rate (eGFR) levels. **(Panel A)** Plasma glucose levels (mg/dL) after 2-hour post-glucose tolerance test (OGTT) (T120’); **(Panel B)** homeostatic model assessment of insulin resistance (HOMA-IR); **(Panel C)** insulin sensitivity index (ISI); **(Panel D)** disposition index (DI); *p* value < 0.05*; < 0.01** after ANOVA corrected for confounding factors (i.e. sex, puberty, BMI).

In 1.4% (5/360) of patients, eGFR was < -1 SD, while in 4 of them it was < -2 SD. Furthermore, eGFR was > 1SD in 60.8% (219/360) of subjects, with 2 of them displaying an eGFR > 2 SD. Anthropometric and metabolic characteristics of subjects according to eGFR categories are reported in Table 2. Compared with subjects with eGFR < -1 SD, patients with eGFR > 1 SD showed higher SBP, AST, ALT, glucose and insulin during OGTT, insulin resistance (Fig 1, Panel A and B); they also had lower sensitivity indexes after both crude analysis and ANCOVA (Fig 1, Panel C and D).

**Table 2:** Anthropometric and metabolic characteristics of the study population according to eGFR stratified for percentiles, age and sex dependent, based on NKF-K/DOQI Guidelines.

		Categories of eGFR				*P* value
		< - 1 SD	- 1–0 SD	0–1 SD	> 1 SD	
**Subjects**		5 (1.4%)	23 (6.4%)	113 (31.4%)	219 (60.8%)	** **
**Age (years)**		11.1±4.0	11.7±3.0	10.7±3.1	10.9±2.9	*ns*
**Sex**	**M**	3 (60%)	5 (21.7%) **	44 (38.9%) ^‡‡^	128 (58.5%)	*< 0*.*0001*
	**F**	2 (40%)	18 (78.3%) **	69 (61.1%) ^‡‡^	91 (41.5%)	
**Puberty**	**PP**	2 (40%)	5 (21.7%)	57 (50.4%)	91 (41.5%)	*ns*
	**P**	3 (60%)	18 (78.3%)	56 (49.6%)	128 (58.5%)	
**BMI (kg/m**^**2**^**)**		30.13±6.74	29.50±5.43	27.50±4.98	28.25±4.06	*ns*
**BMI SDS (kg/m**^**2**^**)**		2.35±0.63	2.21±0.55	2.03±0.48	2.11±0.43	*ns*
**Waist circumference (cm)**		97.1±20.3	93.8±14.1 ^§^	87.5±13.0 ^‡‡^	92.1±13.0	*< 0*.*009*
**Waist/Height ratio**		0.64±0.08	0.63±0.09	0.61±0.08	0.641±0.11	*ns*
**SBP (mmHg)**		135±10	128±15.5	123±16.0 ^‡‡^	128±16.2	*< 0*.*02*
**SBP percentile**		98±1.3	92±9.23	86±17.5 ^‡‡^	91±13.7	*< 0*.*01*
**DBP (mmHg)**		86±4.1	83±10.9	79±11.1	79±10.6	*ns*
**DBP percentile**		98±1.9	92±8.3	87±14.5	86±16.1	*ns*
**Total cholesterol (mg/dL)**		141.8±21.3	138.9±27.5	141.6±25.6	148.5±28.6	*ns*
**HDL-c (mg/dL)**		38.8±8.9	42.3±9.2	43.5±9.4	42.6±8.2	*ns*
**LDL-c (mg/dL)**		86.4±12.5	83.9±26.9	83.4±22.7	89.7±23.6	*ns*
**Triglycerides (mg/dL)**		83.6±54.3	70.5±22.6	74.6±39.6	80.0±46.8	*ns*
**AST (IU/L)**		23.6±10.7	19.5±6.0 ^§^, **	22.9±5.9 ^††^	24.8±7.4	*< 0*.*002*
**ALT (IU/L)**		31.8±25.3	23.7±10.2	20.8±8.3 ^‡‡^	25.9±14.5	*< 0*.*001*
**AST/ALT ratio**		0.86±0.29 ^‡^	0.89±0.31 ^¶^	1.20±0.37 ^‡‡^	1.08±0.39	*< 0*.*001*
**Uric acid (mg/dL)**		4.70±0.43	5.30±0.89	4.72±1.16	4.90±1.24	*ns*
**IGF-1 (ng/mL)**		255.5±102.6	324.4±113.7	280.8±133.1	286.6±130.3	*ns*
**GlcT0' (mg/dL)**		86.6±4.9	87.04±5.75	86.9±7.2	88.5±7.5	*ns*
**GlcT30' (mg/dL)**		152.5±28.0 ^†^	123.5±18.5 ^§, #^	134.2±23.7	135.8±22.3	*< 0*.*0001*
**GlcT60' (mg/dL)**		122.2±36.4	106.7±19.5	111.0±27.2	117.6±25.3	*ns*
**GlcT90' (mg/dL)**		121.0±24.7 ^†^	96.0±16.4 **	104.1±19.17 ^‡‡^	110.8±20.6	*< 0*.*001*
**GlcT120' (mg/dL)**		115.6±21.1	100.0±13.6 **	104.4±17.4 ^‡‡^	110.3±20.6	*< 0*.*009*
AUC Glc (mg/dL*h/dL)		22287.0±17283. 9 ^†^	12614.2±1278.7 **	13393.9.±2008.8 ^††^	13930.7.±1868.0	*< 0*.*004*
**Mean Glc (mg/dL)**		168.8±117.3 *	102.9±9.5 **	108.5±15.0 ^††^	112.6±14.3	*< 0*.*005*
**InsT0' (mUI/L)**		21.7±8.8	13.7±7.1	13.7±9.7 ^‡‡^	17.9±12.3	*< 0*.*006*
**Mean Ins (mUI/L)**		106.0±39.8	60.2±29.7	65.8±47.5	80.9±69.9	*ns*
**HOMA-IR**		4.62±1.92	2.97±1.50	2.95±2.13 ^‡‡^	4.08±3.12	*< 0*.*02*
**ISI**		2.18±1.01	4.24±1.16	6.05±6.85 ^‡‡^	4.18±3.40	*< 0*.*005*
**QUICKI**		0.31±0.01	0.33±0.02	0.35±0.05 ^§§^	0.32±0.04	*< 0*.*001*
**InsI**		2.14±1.27	2.81±3.75	2.20±1.91	2.04±4.79	*ns*
**DI**		5.56±4.48	9.09±9.04	10.30±13.70 ^‡‡^	4.92±18.61	*< 0*.*05*
**u-ACR (mg/g)**		7.35±3.81	4.83±5.18	9.91±18.83	12.78±31.72	*ns*

Data are expressed as mean±SD. *p* value -1SD *vs* -1-0 SD: <0.05*; < 0.01^†^. -1SD *vs* 0-1SD: < 0.05^‡^. -1-0 SD *vs* 0–1 SD: < 0. 05^§^; < 0.0001^¶^; -1-0 SD *vs* > 1SD: < 0.05^#^; < 0.01**. 0–1 SD *vs* > 1SD: < 0.05^††^; < 0.01^‡‡^; < 0.0001^§§^. ns: not significant. ANOVA analysis with a Bonferroni post-hoc test was used. OGTT data are available for 342 or 345 subjects. Abbreviations: ALT: alanine aminotransferase; AST: aspartate aminotransferase; AUC: area under the curve; BMI: body mass index; DI: disposition index; DBP: diastolic blood pressure; eGFR: estimated glomerular filtration rate; F: female; GlcT0’: fasting glucose; GlcT30',T60',T90',T120’: post-challenge glucose; HDL-c: high density lipoprotein cholesterol; HOMA-IR: homeostatic model assessment of insulin resistance; InsI: insulinogenic index; InsT0’: fasting insulin; IOTF: International Obesity Task Force; ISI: insulin sensitivity index; LDL-c: low density lipoprotein cholesterol; M: male; P: pubertal; PP: prepubertal; QUICKI: quantitative insulin-sensitivity check index; SBP: systolic blood pressure; u-ACR: urinary albumin-to-creatinine ratio.

Subjects with an eGFR within 0 and 1 SD had higher odds to have triglycerides < 90^th^ percentile in both crude and controlled models (model 2; OR 1.750; CI 1.002–3.056; p < 0.04). eGFR was correlated with many variables in the crude model. After adjusting for age, sex, puberty, and BMI, eGFR was positively associated with WC, fasting insulin, glucose levels at 90 and 120 minutes, AUC and mean glucose, insulin at 120 minutes, mean insulin and u-ACR, and negatively associated with DPB, uric acid, ISI, and QUICKI. After adjusting for age, sex, puberty, and WC, eGFR was positively associated with fasting insulin, glucose levels at 90 and 120 minutes, AUC and mean glucose, HOMA-IR and u-ACR, and negatively associated with DPB, uric acid, ISI, and QUICKI (Table 3).

**Table 3:** Partial correlations between eGFR and microalbuminuria, uric acid and other cardiometabolic variables.

eGFR	Model 1		Model 2	
	r	*P* value	r	*P* value
**Age (years)**				
**BMI (kg/m**^**2**^**)**			-0.062	*ns*
**BMI SDS (kg/m**^**2**^**)**				
**Waist circumference (cm)**	**0.150**	*< 0*.*005*		
**Waist/Height ratio**				
**SBP (mmHg)**	0.034	*Ns*	0.016	*ns*
**DBP (mmHg)**	**- 0.122**	*< 0*.*002*	**-0.131**	*< 0*.*01*
**Total cholesterol (mg/dL)**	0.092	*Ns*	0.084	*ns*
**HDL-c (mg/dL)**	0.065	*Ns*	0.071	*ns*
**LDL-c (mg/dL)**	0.067	*Ns*	0.066	*ns*
**Triglycerides (mg/dL)**	0.026	*Ns*	-0.002	*ns*
**AST (IU/L)**	0.094	*Ns*	0.085	*ns*
**ALT (IU/L)**	0.037	*Ns*	0.012	*ns*
**AST/ALT ratio**	-0.11	*Ns*	-0.004	*ns*
**Uric acid (mg/dL)**	**-0.172**	*< 0*.*0001*	**-0.217**	*< 0*.*0001*
**IGF-1 (ng/mL)**	0.021	*Ns*	0.042	*ns*
**GlcT0' (mg/dL)**	0.078	*Ns*	0.090	*ns*
**GlcT30' (mg/dL)**	0.032	*Ns*	0.039	*ns*
**GlcT60' (mg/dL)**	0.077	*Ns*	0.083	*ns*
**GlcT90' (mg/dL)**	**0.174**	*< 0*.*001*	**0.176**	*< 0*.*001*
**GlcT120' (mg/dL)**	**0.164**	*< 0*.*002*	**0.168**	*< 0*.*002*
AUC Glc (mg/dL*h/dL)	**0.132**	*< 0*.*01*	**0.128**	*< 0*.*001*
**Mean Glc (mg/dL)**	**0.131**	*< 0*.*01*	**0.140**	*< 0*.*01*
**Mean Ins (mUI/L)**	**0.109**	*< 0*.*04*	0.080	*ns*
**HOMA-IR**	**0.164**	*< 0*.*02*	**0.141**	*< 0*.*009*
**ISI**	**-0.131**	*< 0*.*01*	**-0.108**	*< 0*.*05*
**QUICKI**	**-0.176**	*< 0*.*01*	**-0.140**	*< 0*.*009*
**InsI**	0.009	*Ns*	0.002	*ns*
**DI**	-0.047	*Ns*	-0.041	*ns*
**u-ACR (mg/g)**	**0.124**	*< 0*.*02*	**0.128**	*< 0*.*01*

Model 1: controlled for sex, age, puberty and BMI. Model 2: controlled for sex, age, puberty and waist circumference. ns: not significant. OGTT data are available for 342 or 345 subjects. Abbreviations: ALT: alanine aminotransferase; AST: aspartate aminotransferase; AUC: area under the curve; BMI: body mass index; DI: disposition index; DBP: diastolic blood pressure; eGFR: estimated glomerular filtration rate; GlcT0’: fasting glucose; GlcT30',T60',T90',T120’: post-challenge glucose; HDL-c: high density lipoprotein cholesterol; HOMA-IR: homeostatic model assessment of insulin resistance; InsI: insulinogenic index; InsT0’: fasting insulin; ISI: insulin sensitivity index; LDL-c: low density lipoprotein cholesterol; QUICKI: quantitative insulin-sensitivity check index; SBP: systolic blood pressure; u-ACR: urinary albumin-to-creatinine ratio.

### Hyperuricemia evaluation and its association with microalbuminuria, eGFR and other cardiometabolic variables

Hyperuricemia was present in 27.5% (99/360) of subjects, of whom 8.1% (8/99) had microalbuminuria, whereas microalbuminuria was found in 5.7% (15/261) of subjects without hyperuricemia. Interestingly, 64.6% (64/99) of subjects with hyperuricemia had eGFR > 1 SD, and 26.3% (26/99) were between 0 and 1 SD. In contrast, no subject with eGFR < -1 SD had hyperuricemia. Conversely, 59.4% (155/261) of subjects without hyperuricemia had eGFR > 1 SD, and 33.3% (87/261) were between 0 and 1 SD.

Subjects with hyperuricemia were older, had higher BMI, BMI SDS, waist circumference, SBP, DBP, triglycerides, ALT and IGF-1 levels and lower HDL-cholesterol, AST, AST to ALT ratio, eGFR and u-ACR compared with those with normal acid uric levels. Moreover, subjects with hyperuricemia showed higher glucose and insulin, either at fasting or as responses to OGTT, associated with higher insulin resistance and lower insulin sensitivity than those without hyperuricemia. After controlling for confounding factors, subjects with hyperuricemia maintained higher levels of IGF-1 and lower eGFR compared to those with normal acid uric levels (Table 4). Uric acid levels were positively associated with ALT, IGF-1, HOMA-IR, fasting insulin, glucose levels at 60, 90 and 120 minutes, AUC and mean glucose, insulin at 60 minutes, and negatively associated with HDL-cholesterol, AST to ALT ratio, eGFR, and QUICKI, also when corrected for covariates (Table 5).

**Table 4:** Anthropometric and metabolic characteristics of the study population according to uric acid.

	URIC ACID *P* value			
	Normal	High	Model 1	Model 2
			
**Age (years)**	10.2±3.0	12.7±2.5		
**BMI (kg/m**^**2**^**)**	27.1±4.5	30.8±4.7		*ns*
**BMI SDS (kg/m**^**2**^**)**	2.03±0.46	2.27±0.54		*ns*
**Waist circumference (cm)**	87.5±13.4	100.3±12.4	*< 0*.*0001*	
**Waist/Height ratio**	0.62±0.10	0.64±0.12	*ns*	
**SBP (mmHg)**	123.2±16.1	134.5±17.7	*< 0*.*01*	*ns*
**SBP percentile**	88.7±15.0	92.1±13.7	*< 0*.*01*	*ns*
**DBP (mmHg)**	77.8±10.7	84.2±10.8	*< 0*.*05*	*ns*
**DBP percentile**	85.6±16.2	90.2±12.4	*< 0*.*05*	*ns*
**Total cholesterol (mg/dL)**	146.9±28.1	142.1±26.4	*ns*	*ns*
**HDL-c (mg/dL)**	43.8±8.7	40.1±7.9	*ns*	*ns*
**LDL-c (mg/dL)**	88.2±23.8	84.8±22.8	*ns*	*ns*
**Triglycerides (mg/dL)**	74.6±43.5	86.1±49.7	*ns*	*ns*
**AST (IU/L)**	24.2±7.0	22.9±6.6	*ns*	*ns*
**ALT (IU/L)**	23.1±13.1	27.5±16.6	*ns*	*ns*
**AST/ALT ratio**	1.16±0.39	0.95±0.30	*ns*	*ns*
**Uric acid (mg/dL)**	4.3±0.8	6.3±0.7	*ns*	*ns*
**IGF-1 (ng/mL)**	258.6±115.5	353.8±132.4	*< 0*.*04*	*< 0*.*03*
**eGFR (mL/min/1.73m**^**2**^**)**	121.8±19.4	113.6±18.5	*ns*	*< 0*.*001*
**25-OH VitD (ng/mL)**	20.3±9.2	20.3±9.6	*ns*	*ns*
**GlcT0' (mg/dL)**	87.3±8.9	90.4±12.3	*ns*	*ns*
**GlcT30' (mg/dL)**	135.3±23.5	133.2±20.6	*ns*	*ns*
**GlcT60' (mg/dL)**	111.7±25.8	123.2±26.4	*ns*	*ns*
**GlcT90' (mg/dL)**	106.4±20.7	112.6±22.0	*ns*	*ns*
**GlcT120' (mg/dL)**	106.6±18.7	111.8±19.7	*ns*	*ns*
AUC Glc (mg/dL*h/dL)	1370.2±2870.7	14109.1±1943.1	*ns*	*ns*
**Mean Glc (mg/dL)**	110.7±20.6	114.3±14.9	*ns*	*ns*
**InsT0' (mUI/L)**	146.1±13.1	22.3±18.9	*< 0*.*01*	*ns*
**InsT30' (mUI/L)**	123.6±186.2	108.4±80.8	*ns*	*ns*
**InsT60' (mUI/L)**	80.0±86.7	109.7±104.8	*ns*	*ns*
**InsT90' (mUI/L)**	68.9±65.2	86.6±75.2	*ns*	*ns*
**InsT120' (mUI/L)**	71.2±92.7	100.5±126.3	*ns*	*ns*
AUC Ins (mUI*h/dL)	9508.7±8260.3	10988.4±7915.6	*ns*	*ns*
**Mean Ins (mUI/L)**	72.9±62.6	85.6±64.2	*ns*	*ns*
**HOMA-IR**	3.27±4.25	5.33±6.84	*< 0*.*05*	*ns*
**ISI**	5.13±4.79	3.66±2.91	*ns*	*ns*
**QUICKI**	0.34±0.04	0.32±0.05	*ns*	*ns*
**InsI**	2.17±4.50	2.06 ±2.50	*ns*	*ns*
**DI**	7.25±6.84	5.62±6.18	*ns*	*ns*
**u-ACR (mg/g)**	11.59±26.93	10.53±24.50	*ns*	*ns*

Data are expressed as mean±SD. ANCOVA Model 1: controlled for sex, age, puberty and BMI. ANCOVA Model 2: controlled for sex, age, puberty and waist circumference. ns: not significant. OGTT data are available for 342 or 345 subjects. Abbreviations: ALT: alanine aminotransferase; AST: aspartate aminotransferase; AUC: area under the curve; BMI: body mass index; DI: disposition index; DBP: diastolic blood pressure; eGFR: estimated glomerular filtration rate; F: female; GlcT0’: fasting glucose; GlcT30',T60',T90',T120’: post-challenge glucose; HDL-c: high density lipoprotein cholesterol; HOMA-IR: homeostatic model assessment of insulin resistance; InsI: insulinogenic index; InsT0’: fasting insulin; InsT30',T60',T90',T120': post-challenge glucose; ISI: insulin sensitivity index; LDL-c: low density lipoprotein cholesterol; M: male; QUICKI: quantitative insulin-sensitivity check index; SBP: systolic blood pressure; u-ACR: urinary albumin-to-creatinine ratio; uric acid: high (≥ 5.5 mg/dL); 25OH VitD: 25-0H vitamin D.

**Table 5:** Partial correlations between uric acid and microalbuminuria, eGFR and other cardiometabolic variables.

URIC ACID	Model 1		Model 2	
	r	*P* value	r	*P* value
**Age (years)**		* *		
**BMI (kg/m**^**2**^**)**			0.015	*ns*
**BMI SDS (kg/m**^**2**^**)**				
**Waist circumference (cm)**	**0.169**	*< 0*.*002*		
**Waist/Height ratio**				
**SBP (mmHg)**	**0.105**	*< 0*.*05*	0.078	*ns*
**DBP (mmHg)**	0.076	*ns*	0.088	*ns*
**Total cholesterol (mg/dL)**	0.052	*ns*	0.044	*ns*
**HDL-c (mg/dL)**	**-0.153**	*< 0*.*004*	**-0.150**	*< 0*.*005*
**LDL-c (mg/dL)**	0.081	*ns*	0.079	*ns*
**Triglycerides (mg/dL)**	**0.105**	*< 0*.*04*	0.087	*ns*
**AST (IU/L)**	0.018	*ns*	0.009	*ns*
**ALT (IU/L)**	**0.115**	*< 0*.*03*	**0.104**	*< 0*.*05*
**AST/ALT ratio**	-0.118	*< 0*.*02*	**-0.122**	*< 0*.*02*
**IGF-1 (ng/mL)**	**0.187**	*< 0*.*0001*	**0.217**	*< 0*.*0001*
**eGFR (mL/min/1.73m**^**2**^**)**	**-0.172**	*< 0*.*001*	**-0.217**	*< 0*.*0001*
**GlcT0' (mg/dL)**	0.070	*ns*	0.074	*ns*
**GlcT30' (mg/dL)**	0.096	*ns*	0.089	*ns*
**GlcT60' (mg/dL)**	**0.209**	*< 0*.*0001*	**0.206**	*< 0*.*0001*
**GlcT90' (mg/dL)**	**0.118**	*< 0*.*03*	**0.111**	*< 0*.*04*
**GlcT120' (mg/dL)**	**0.137**	*< 0*.*01*	**0.136**	*< 0*.*01*
AUC Glc (mg/dL*h/dL)	**0.176**	*< 0*.*001*	**0.168**	*< 0*.*002*
**Mean Glc (mg/dL)**	**0.179**	*< 0*.*001*	**0.178**	*< 0*.*001*
**InsT0' (mUI/L)**	**0.181**	*< 0*.*001*	**0.014**	*< 0*.*009*
**Mean Ins (mUI/L)**	**0.114**	*< 0*.*003*	0.072	*ns*
**HOMA-IR**	**0.140**	*< 0*.*008*	**0.117**	*< 0*.*03*
**ISI**	**-0.120**	*< 0*.*02*	-0.096	*ns*
**QUICKI**	**-0.150**	*< 0*.*0001*	**-0.113**	*< 0*.*03*
**InsI**	-0.037	*ns*	-0.049	*ns*
**DI**	-0.090	*ns*	-0.091	*ns*
**u-ACR (mg/g)**	-0.085	*ns*	-0.080	*ns*

Model 1: controlled for sex, age, puberty and BMI. Model 2: controlled for sex, age, puberty and waist circumference. ns: not significant. Abbreviations: ALT: alanine aminotransferase; AST: aspartate aminotransferase; AUC: area under the curve; BMI: body mass index; DI: disposition index; DBP: diastolic blood pressure; eGFR: estimated glomerular filtration rate; GlcT0’: fasting glucose; GlcT30',T60',T90',T120’: post-challenge glucose; HDL-c: high density lipoprotein cholesterol; HOMA-IR: homeostatic model assessment of insulin resistance; InsI: insulinogenic index; InsT0’: fasting insulin; ISI: insulin sensitivity index; LDL-c: high density lipoprotein cholesterol; QUICKI: quantitative insulin-sensitivity check index; SBP: systolic blood pressure; u-ACR: urinary albumin-to-creatinine ratio.

Lastly, hyperuricemia was associated with hypertension (OR: 2.086, CI 1.257–3.460, p < 0.003), HDL-cholesterol ≤ 10^th^ percentile (OR: 2.001, CI 1.252–3.198; p < 0.003) and glucose ≥ 155.0 mg/dL at 60 minutes after OGTT (OR: 2.350, CI 1.045–5.282; p < 0.03) in both crude and controlled models.

## Discussion

This study shows a worsened metabolic profile in obese children with normal eGFR higher than 1 SD or with abnormally high serum uric acid levels. In particular, post-OGTT glucose levels were found to be higher, albeit within standard cutoffs, in those individuals with eGFR > 1 SD. Furthermore, subjects with microalbuminuria did not display a major impairment in their cardiometabolic alterations, although all of them had eGFR > 0 SD.

In recent years, a positive correlation between microalbuminuria and obesity in adult patients has been clearly established [3]. However, in pediatric patients such correlation appears to be less obvious and only partially understood probably due to the lack of consistent data on large-sized cohorts. In this regard, here we show a prevalence of 6.4% of microalbuminuria in a large cohort of obese children and adolescents, which is in good agreement with previous data reporting prevalence of microalbuminuria ranging between 0.3% and 10.1% in similar patient groups [16–21]. These studies, including ours, however appears to underestimate the prevalence of microalbuminuria when compared to other reports showing a much higher prevalence of microalbuminuria in obese children, which could go as high as 37.0% [22, 23]. This discrepancy could be explained by different modalities of urine sample collection [22] or by the measurement of urinary albumin excretion rate per minute time rather than u-ACR [23]. Moreover, other variables such as postural changes and exercise before the testing session, as well as ethnicity, might account for data variability [24], even though no evidence indicating that this is indeed the case in obese children has been found in previous studies [18, 19].

Associations between microalbuminuria and other cardiometabolic markers in obese children and adolescents have been reported in some [16, 18, 20, 21, 22, 25] but not all studies [17, 19, 26], including ours. Although this lack of correlation could be explained by the young mean age of our patients or the low prevalence of microalbuminuria registered in these latter, our findings do not support a routine assessment of microalbuminuria in all obese children and adolescents.

Interestingly, we find a lower insulinogenic index in subjects with microalbuminuria, which is in good agreement with a recent study demonstrating a positive correlation between microalbuminuria and HbA1c in obese Korean adolescents [21]. In this regard, it has been hypothesized that the prevalence of microalbuminuria progressively increased as plasma glucose values climbed through the ‘normal range’ into the impaired range of glucose tolerance, suggesting that the effect of glucose may be continuous. Furthermore, frequent daily postprandial states of relatively higher glucose levels could increase oxidative stress on the vessels leading to increased urinary albumin excretion secondary to endothelial dysfunction [16]. Moreover, because HbA1c and insulinogenic-index are both related to insulin-resistance, also the latter could be a responsible for this alteration. This is in agreement with the evidence that insulin-resistance contributes to micro and macrovascular disease [1, 16].

Since our results appear to indicate that subjects with eGFR > 0 SD have microalbuminuria and a suboptimal metabolic phenotype, further longitudinal studies on larger populations are clearly needed to fully establish whether eGFR could be used as a useful marker to stratify high-risk obese youths.

According to NKF-K/DOQI guidelines [13], we also report eGFR < -1 SD in 1.4% of our population, who was free of known CKDs. Based on a normal population distribution, a much greater percentage would be expected to be below -1 SD and again below normal -2 SD. The fact that the majority of our subjects were stratified above 0 and, to a greater extent, 1 SD indicates a quite alarming skewed distribution of eGFR in obesity, in agreement with some [27], but not all authors [20, 28]. In fact, differently by us, the distribution observed in the National Health and Nutrition Examination Survey (NHANES) resulted toward lower eGFR beyond what is expected. However, the NHANES cohort included only adolescents aged 12–19 years of general U.S. population [28], while our population was much younger. These contrasting findings could be due also to different obesity trajectories or different formulas used to determine eGFR from serum creatinine concentration (i.e Jaffè technique *vs* Schwartz’s formula) [14]. Therefore, previous reports have clearly shown that adulthood obesity is associated with glomerular hyper-perfusion and hyper-filtration as an early sign of physiologic maladaptation leading, in part, to afferent arteriolar vasodilatation [1]. Of note, we show that microalbuminuria was present only in subjects with eGFR > 0 SD in good agreement with a previous report [29]. Thus, our findings, together with the observation that childhood obesity positively correlates with a fast decline of eGFR over time, with a 2- to -3 fold higher risk of developing ESRD [30], should prompt physicians to evaluate the possibility of renal dysfunction in obese children.

We also show that subjects with eGFR > 1 SD presented with an increased burden of cardiometabolic alterations as recent studies in adults seem to suggest [2]. In particular, in our young patients with eGFR > 1 SD, systolic blood pressure, glucose, and insulin levels in response to OGTT and insulin resistance were higher, whereas insulin sensitivity was lower compared to other subgroups, suggesting a glucose dysregulation mainly after OGTT. The same condition was also present in patients with eGFR < -1 SD, suggesting a U-shaped relationship, even though the low number of subjects in the left part of the curve does not allow us to draw any firm conclusions about its generalizability. A similar U-shaped effect between eGFR, blood pressure and microalbuminuria has been recently reported by Di Bonito P. et al. [29], although these authors could not find a significant relationship between eGFR and the glyco-insulinemic profile at fasting. This discrepancy might be due to the different eGFR cut-off in adults used in this study.

Overall, our findings raise the possibility that eGFR > 1 SD may be an early predictor of dysglycemia and pre-diabetes, a possibility that could be further explored by investigating the relationships among post-challenge glucose, insulin levels and kidney function in youths. Our data are also consistent with findings by Matsushita et al., who have recently shown that the inclusion of eGFR and u-ACR among traditional risk predictors greatly improved the discrimination of cardiometabolic outcomes in adults [4]. However, additional longitudinal studies are clearly needed to establish the evolution and distribution of eGFR in obese children and explain its pathophysiological significance over time.

In our study, we show an inverse correlation between eGFR and uric acid, which is in line with previous reports on several adult populations [6] and adolescents with type 1 diabetes [31]. While in the past hyperuricemia was thought to result from a decreased uric acid clearance due to kidney damage, it now seems that uric acid *per se* might play a role in the natural history of GFR decline [6]. In this regard, here we show that hyperuricemic patients are at increased risk of having a 1-hour post-OGTT glycemia ≥ 155.0 mg/dL. This cut-off seems to be associated with an increased metabolic risk in subjects with a post-challenge normal glucose tolerance as well as with the development of an overt type 2 diabetes rather than fasting glucose [32]. Moreover, serum uric acid levels are closely related to both early-phase insulin secretion and 2-hour post-challenge glucose levels in adults with apparently normal glucose regulation [33].

To the best of our knowledge, this is the first study in a pediatric obese population where a positive association between uric acid and glucose response after OGTT has been found. In line with previous evidence [5], we confirm a worse cardiometabolic profile in subjects with hyperuricemia, which increased the odds of hypertension and HDL-cholesterol ≤ 10^th^ percentile. In agreement with recent studies in adults [34], we also observed an association between uric acid and ALT, which suggests that uric acid may be an independent risk factor for liver diseases.

Although serum uric acid seems a good predictor of renal and cardiometabolic diseases, its normal values in children and adolescents are still undefined. In this regard, we report an age-dependent effect on serum uric acid levels. Thus, the fixed cut-off is probably improper in the pediatric population, while a distribution according to age and sex may be more appropriate.

All in all, our results are limited to a Caucasian population. We included only Caucasian children and adolescents because ethnic influences on microalbuminuria and serum uric acid have been reported [18, 19, 24, 35]. Therefore, further studies on more heterogeneous populations are needed.

Our study has some limitations. First of all, the cross-sectional design does not allow us to conclude that there is a causal relationship between variables; longitudinal studies might clarify this aspect. Moreover, a normal-weight control group is lacking, because the study was performed in a tertiary referral center. Another limit is that microalbuminuria was measured on spot morning urine samples; however, spot u-ACR correlates very well with the urine collection at the 24-hour time point [19]. Furthermore, we failed to observe a normal distribution of eGFR in obesity, and a very low percentage of subjects could be stratified in the extreme tails (±2SD). Thus, studies on larger populations are needed to confirm our data and investigate the metabolic phenotype of those with an eGFR above or below ±2SD.

On the other hand, our study includes a large sample of subjects as well as the availability of OGTT for the majority of patients. Moreover, microalbuminuria was confirmed on three samples, and eGFR was stratified according to the pediatric cut-off, unlike most publications on pediatric obesity.

In conclusion, our study suggests that eGFR may be helpful in clinical practice to identify an unhealthy metabolic profile in pediatric obesity. Thus, more attention should be paid to this relatively inexpensive parameter. Therefore, in subjects with an eGFR > 1 SD or hyperuricemia, we encourage to investigate the early-phase insulin secretion and 2-hour post-challenge glucose levels. Serum uric acid seems to be another useful tool to diagnose subjects at high risk of metabolic impairment. However, studies based on larger population are needed to establish normal references values according to age and sex.

Finally, based on our data, we strongly recommend the inclusion of microalbuminuria only in routine screenings of pediatric obese patients with eGFR greater than 1 SD. Further studies on large-sized pediatric cohorts are needed to confirm our finding also in obese children with an eGFR less than -2 SD or greater than 2 SD.

## Supporting information

S1 Table STROBE checklist. (DOCX)

## Abbreviations

AST: Aspartate aminotransferase
ALT: Alanine aminotransferase
AUC: Area under the curve
BMI: Body mass index
BMI-SDS: BMI standard deviation score
DBP: Diastolic blood pressure
CDK: Chronic kidney disease
DI: Disposition index
eGFR: Estimated glomerular filtration rate
ESRD: End-stage renal disease
HDL: High density lipoprotein cholesterol
(HOMA)-IR: Homeostasis model assessment–insulin resistance
IOTF: International Obesity Task Force
InsI: Insulinogenic index
ISI: Matsuda index
LDL: Low-density lipoprotein -cholesterol
LMS: Least median squares method
NHANES: National Health and Nutrition Examination Survey
OR: Odds ratio
OGTTs: Oral glucose tolerance tests
QUICKI: Quantitative Insulin-Sensitivity Check Index
SBP: Systolic blood pressure
SD: Standard deviation
u-ACR: Urinary albumin-to-creatinine ratio
WC: Waist circumference
