# Supplementary material for: High-normal estimated glomerular filtration rate and hyperuricemia positively correlate with metabolic impairment in pediatric obese patients
Source: PLoS One. 2018 Mar 5;13(3):e0193755. doi: 10.1371/journal.pone.0193755 (PMC5837119; doi:10.1371/journal.pone.0193755)
Supplement: S1 Table — (DOCX) [file pone.0193755.s001.docx]

**Supporting Table 1**

**STROBE Checklist**

**Title and Abstract**

*1a. Indicate the study’s design with a commonly used term in the Title or the Abstract.*

This has been done in Abstract Section. The study was a cross sectional population based study.

*1b.Provide in the abstract an informative and balanced summary of what was done and what was found.*

This has been done with explanation of main outcomes and principal results (pag.3).

**Introduction**

*2. Background/rationale. Explain the scientific background and rationale for the investigation being reported.*

This has been done in the Introduction Section. See pag. 4.

*3. Objectives. State specific objectives, including any prespecified hypotheses.*

This has been done. Aims has been subdivided in several points. See pag. 4-5.

**Methods**

*4. Study design. Present key elements of study design early in the paper.*

This has been done in the Subsection “Study design” (pag. 6)

*5. Setting. Describe the setting, locations, and relevant dates, including periods of recruitment, exposure, follow-up, and data collection.*

All these data have been included in the subsection “study design” (pag. 6).

*6. Participants. Cross sectional study*—*Give the eligibility criteria, and the sources and methods of selection of participants.*

All these data have been included in the Subsections “Study design” “Anthropometric, biochemical measurements and definitions” (pag. 6).

*7. Variables. Clearly define all outcomes, exposures, predictors, potential confounders, and effect modifiers. Give diagnostic criteria, if applicable.*

All these data have been included in the Subsections “Anthropometric, biochemical measurements and definitions” (pag. 6).

*8. Data source/measurements. For each variable of interest, give sources of data and details of methods of assessment (measurement). Describe comparability of assessment methods if there is more than one group.*

All these data have been included in the Subsections “Anthropometric, biochemical measurements and definitions” (pag. 6) and in “Suppl. Material II”.

*9. Describe any efforts to address potential sources of bias.*

Possible bias have been considered in Conclusion Section about study limitations (pag. 17).

*10. Study size. Explain how the study size was arrived at.*

These data have been included in the Subsections “Study design” (pag. 6) and in “Statistical analysis (pag. 9).

*11. Explain how quantitative variables were handled in the analyses. If applicable, describe which groupings were chosen and why.*

How quantitative variable were handled was explained in the Subsection “Statistical analysis” (pag. 7). The choice of subgroups was explained at Subsections “Study design” and “Anthropometric, biochemical measurements and definitions” (pag. 6).

*12. Statistical methods (a-e).*

All information have been provided in the Subsection “Statistical analysis” (pag. 9).

**Results**

*13. (a) Report numbers of individuals at each stage of study—eg numbers potentially eligible, examined for eligibility, confirmed eligible, included in the study, completing follow-up, and analysed. (b) Give reasons for non-participation at each stage(c) Consider use of a flow diagram.*

All these data have been included in Results, in particular in the Subsection “Anthropometric and metabolic characteristics” (pag. 11). Nobody refused the study. Flow diagram was not necessary because it was an one step cross sectional observational study.

*14. Descriptive data. (a) Give characteristics of study participants (eg demographic, clinical, social) and information on exposures and potential confounders (b) Indicate number of participants with missing data for each variable of interest (c) Cohort study—Summarize follow-up time (eg average and total amount).*

All these data have been included in Results, in particular in the Subsection “Anthropometric and metabolic characteristics” and in Table 1. Follow-up time was not described because of the cross sectional nature of the study.

15. *Outcome data. Cross sectional study—Report numbers of outcome events or summary measures.*

Measure have been summarized in subheadings.

16. *Main results. (a) Report the numbers of individuals at each stage of the study—eg numbers potentially eligible, examined for eligibility, confirmed eligible, included in the study, completing follow-up, and analyzed (b) Give reasons for non-participation at each stage (c) Consider use of a flow diagram*

All these data have been included in Results and Tables. Nobody refused the consent. Flow diagram was not necessary because it was an one step cross sectional observational study. However, each main outcome was inserted in a subheading to assist for interpretation.

**Discussion**

18. *Key results. Summarise key results with reference to study objectives.*

Key results have been summarised (see pag. 14). Each objective of the study have been discussed.

19. *Limitations. Discuss limitations of the study, taking into account sources of potential bias or imprecision. Discuss both direction and magnitude of any potential bias.*

Limitations have been discussed at pag. 17 in a specific paragraph.

20. *Interpretation. Give a cautious overall interpretation of results considering objectives, limitations, multiplicity of analyses, results from similar studies, and other relevant evidence.*

A cautious interpretation considering other studies has been given for each specific aim.

21.*Generalisability. Discuss the generalisability (external validity) of the study results.*

Generalisability has been discussed at each specific point.

**Other information**

22.*Funding. Give the source of funding and the role of the funders for the present study and, if applicable, for the original study on which the present article is based.*

Funding has been described in a specific point at pag. 2.
